# Various facets of intermolecular transfer of phase coherence by nuclear dipolar fields

**DOI:** 10.5194/mr-4-271-2023

**Published:** 2023-12-21

**Authors:** Philippe Pelupessy

**Affiliations:** Laboratoire des Biomolécules, LBM, Département de Chimie, École Normale Supérieure, PSL University, Sorbonne Université, CNRS, 75005 Paris, France

## Abstract

It has long been recognized that dipolar fields can mediate intermolecular transfer of phase coherence from abundant solvent to sparse solute spins. Generally, the dipolar field has been considered while acting during prolonged free-precession delays. Recently, we have shown that transfer can also occur during suitable uninterrupted radio frequency pulse trains that are used for total correlation spectroscopy. Here, we will expand upon the latter work. First, analytical expressions for the evolution of the solvent magnetization under continuous irradiation and the influence of the dipolar field are derived. These expressions facilitate the simulations of the transfer process. Then, a pulse sequence for the retrieval of high-resolution spectra in inhomogeneous magnetic fields is described, along with another sequence to detect a transfer from an intermolecular double-quantum coherence. Finally, various schemes are discussed where the magnetization is modulated by a combination of multiple selective radio frequency pulses and pulsed field gradients in different directions. In these schemes, the magnetization is manipulated in such a way that the dipolar field, which originates from a single-spin species, can be decomposed into two components. Each component originates from a part of the magnetization that is modulated in a different direction. Both can independently, but simultaneously, mediate an intermolecular transfer of phase coherence.

## 1 Introduction

In liquid-state NMR, the magnetization of an abundant or a highly polarized spin species affects the evolution of the density operator through radiation damping (RD) [Bibr bib1.bibx30] and through the dipolar field (DF) [Bibr bib1.bibx10]. RD stems from the radio frequency (rf) field caused by the current that the transverse magnetization induces in an rf coil [Bibr bib1.bibx3]. The DF describes the direct contributions of the longitudinal and transverse nuclear magnetization components to the static field $B_{0}$ and to a perpendicular rf field $B_{1}$ , respectively, through dipolar interactions. It is also known as the DDF, which, in older works, stood for the dipolar demagnetizing field [Bibr bib1.bibx9] but, in more recent works, has been redefined as the distant dipolar field [Bibr bib1.bibx1]. Both RD and the DF can be incorporated into a modified set of Bloch equations [Bibr bib1.bibx4]. Since RD results in a field with only a rapidly oscillating transverse component, its effect on other resonances is usually limited to nearby frequencies. When the chemical shift differences are removed from the effective Hamiltonian by suitable pulse sequences, the effects of RD extend over a much wider range of frequencies [Bibr bib1.bibx24]. Conversely, the DF has a longitudinal component which causes a shift in the precession frequencies of all nuclei that possess a spin [Bibr bib1.bibx11]. Striking effects are observed when the magnetization of an abundant spin species depends on its spatial position, often as a result of a pulsed field gradient (PFG). These non-trivial effects include multiple spin echoes in two-pulse experiments [Bibr bib1.bibx2] and intermolecular multiple quantum cross-peaks in COSY-like (correlation spectroscopy) sequences. These peaks can stem from like [Bibr bib1.bibx15] or unlike [Bibr bib1.bibx32] spins. The phase coherence of abundant spins can also be transferred by the DF during pulse trains that are commonly used in homo-nuclear total correlation spectroscopy (TOCSY) [Bibr bib1.bibx25]. As with RD in these type of experiments, the small transverse component of the DF plays an important role even if chemical shift differences are large.

In this work, several aspects of the transfer that is mediated by the DF and that occurs during continuous pulse trains will be explored: broadband in-phase transfer and high-resolution spectra can be obtained in inhomogeneous $B_{0}$ fields in a fashion similar to the HOMOGENIZED (homogeneity enhancement by intermolecular zero-quantum detection; [Bibr bib1.bibx31]) and related experiments [Bibr bib1.bibx22]. In addition, a transfer of phase coherence can be realized from intermolecular double-quantum (DQ) coherences involving the abundant solvent and the sparse solute spins. Finally, experiments where the magnetization is modulated in more complex ways by applying several PFGs in combination with selective rf pulses will be discussed.

**Figure 1 Ch1.F1:**
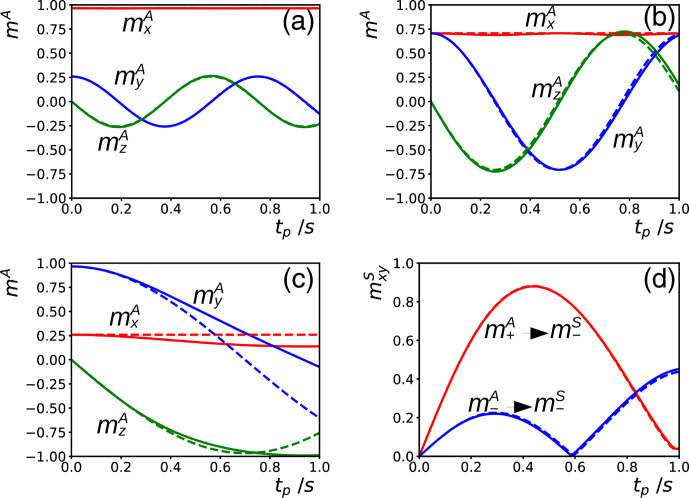
**(a–c)** Simulations of the evolution of the magnetization of the abundant spins A during a time $t_{p}$ of an on-resonance DIPSI-2 pulse train for different initial conditions corresponding to magnetization in the xy plane at angles of 15 ${}^{\circ }$ **(a)**, 45 ${}^{\circ }$ **(b)**, and 75 ${}^{\circ }$ **(c)** with respect to the x axis. The amplitude of the rf field was $\omega_{1}/2\pi =8.33$ kHz, and the DF was characterized by $\omega_{d}=2\pi \times 1.84$ rad s ${}^{-1}$ . The magnetization is recorded stroboscopically after each full cycle. The solid lines result from numerical integration of the modified Bloch Eq. ([Disp-formula Ch1.E2]), as in [Bibr bib1.bibx25]. The dashed lines correspond to Eq. ([Disp-formula Ch1.E3]), with $\omega_{1x}=0$ . In the supporting information, similar simulations with GARP irradiation are shown. For continuous unmodulated irradiation or a WALTZ-16 pulse train, no significant differences appear between the two different ways of calculating the evolution of the magnetization (i.e., the results of either method follow the dashed curves in this figure). **(d)** Simulation of the transfer of phase coherence from spins A to S with the experiment described in [Bibr bib1.bibx25]: a selective excitation of the A spins followed by a DIPSI-2 pulse train bracketed by two PFGs of equal area which have either equal (to observe a transfer $m_{+}^{A}\to m_{-}^{S}$ ) or opposite signs ( $m_{-}^{A}\to m_{-}^{S}$ ). The parameters for the simulation were the same as in **(a)**–**(c)**. The difference in chemical shift between the A and S spins was set to - 3806 Hz. Similarly to **(a)**–**(c)**, the trajectory of the magnetization of the A spins at each position has been obtained either by numerical integration (leading to the solid lines) or by neglecting the DF within each DIPSI-2 cycle while using the approximate solution of Eq. ([Disp-formula Ch1.E3]) for the global evolution between the cycles (dashed lines). We assumed the gradients to be linear.

## 2 Theory

### 2.1 The evolution of the magnetization during rf pulse trains

The following theory, originally developed by [Bibr bib1.bibx9], applies to a homo-nuclear spin system that contains an abundant-spin species A and a sparse-spin species S (both having a spin of 1/2 ), where the magnetization of the spins A has been modulated in a manner that it averages out over the effective sample volume ([Bibr bib1.bibx33], expanded the theory for the case where this condition is not met). Moreover, the spatial variations must be in a single direction s . These modulations are usually induced by a field gradient which is oriented at an angle $\theta_{G}$ with respect to $B_{0}$ , by convention along the z axis, so that $\mathrm{cos}\theta_{G}=\hat{s}\cdot \hat{z}$ . The DF can then be characterized by an angular frequency $\omega_{d}$ , defined as follows:

1 $$\omega_{d}=\frac{1}{3}\mu_{0}\gamma M_{\mathrm{eq}}^{A}\left(3{\mathrm{cos}}^{2}\theta_{G}-1\right),$$

with γ being the gyro-magnetic ratio of the A and S spins, $\mu_{0}$ being the vacuum permeability, and $M_{\mathrm{eq}}^{A}$ being the magnitude of the magnetization of the A spins at equilibrium. In the rotating frame, the evolution of the magnetization vectors of both A and S spins is governed by the modified Bloch equations [Bibr bib1.bibx9]:

2 $$\begin{array}{rl} {\dot{m}}_{x}^{i}= & -\left(\omega_{0}^{i}+\omega_{d}m_{z}^{A}\right)m_{y}^{i}\;+\;\left(\omega_{1y}-\omega_{d}m_{y}^{A}/2\right)m_{z}^{i}\;,\\ {\dot{m}}_{y}^{i}= & \left(\omega_{0}^{i}+\omega_{d}m_{z}^{A}\right)m_{x}^{i}\;-\;\left(\omega_{1x}-\omega_{d}m_{x}^{A}/2\right)m_{z}^{i}\;,\\ {\dot{m}}_{z}^{i}= & -\left(\omega_{1y}-\omega_{d}m_{y}^{A}/2\right)m_{x}^{i}\\ & +\;\left(\omega_{1x}-\omega_{d}m_{x}^{A}/2\right)m_{y}^{i}\;, \end{array}$$

with i being either A or S , $\omega_{1x/y}$ being the (time-dependent) rf field, and $\omega_{0}$ being the offset from the carrier frequency. The magnetization components, written in lower-case m , are normalized with respect to the equilibrium amplitudes. These equations are local (as a result of the modulations being only in one direction; [Bibr bib1.bibx9]) so that the evolution can be calculated separately for each position in the sample. Typically, the rf term does not appear in these equations since, previously, the DF has been considered predominantly during free-precession delays. The explicit position and time dependence of the variables have been omitted in these equations. A set time t=T will be indicated in brackets. Neither relaxation nor molecular motions by diffusion or convection have been taken into account.

The set of coupled equations in Eq. ([Disp-formula Ch1.E2]) is non-linear when i=A , while for the sparse spins i=S , the magnetization of the A spins $m^{A}=(m_{x}^{A},m_{y}^{A},m_{z}^{A})$ is the source of a time-dependent field $\gamma B_{d}=(-\omega_{d}m_{x}^{A}/2,-\omega_{d}m_{y}^{A}/2,\omega_{d}m_{z}^{A})$ . Hence, the evolution of the magnetization of the S spins $m^{S}=(m_{x}^{S},m_{y}^{S},m_{z}^{S})$ can be calculated straightforwardly from the trajectory of $m^{A}$ . In [Bibr bib1.bibx25], the evolution of $m^{A}$ during the rf pulse trains has been obtained by numerical integration of the non-linear set of Eq. ([Disp-formula Ch1.E2]). In this work, a DIPSI-2 (decoupling in presence of scalar interactions; [Bibr bib1.bibx27]) pulse train is always applied at the resonance frequency of the A spins, in which case the trajectory of $m^{A}$ can be approximated analytically as follows: in Appendix A, it is derived that, for a strong constant on-resonance rf field ( $\left|\omega_{1}|\gg |\omega_{d}\right|$ ) along the x axis, $m^{A}$ rotates around x with an angular frequency of $\omega_{1x}-3m_{x}^{A}(0)\omega_{d}/4$ :

3 $$\begin{array}{rl} m_{x}^{A} & =m_{x}^{A}(0),\\ m_{y}^{A} & =\mathrm{cos}\left\{\omega_{1x}t-3\omega_{d}m_{x}^{A}(0)t/4\right\}m_{y}^{A}(0)\\ & -\mathrm{sin}\left\{\omega_{1x}t-3\omega_{d}m_{x}^{A}(0)t/4\right\}m_{z}^{A}(0),\\ m_{z}^{A} & =\mathrm{cos}\left\{\omega_{1x}t-3\omega_{d}m_{x}^{A}(0)t/4\right\}m_{z}^{A}(0)\\ & +\mathrm{sin}\left\{\omega_{1x}t-3\omega_{d}m_{x}^{A}(0)t/4\right\}m_{y}^{A}(0). \end{array}$$

Many sequences used for decoupling or magnetization transfer consist of a repetitive cycle of phase-alternated pulses along one axis (in this work, this is assumed to be between +x and -x ), as is the case for, for example, DIPSI-2, Waltz-16 [Bibr bib1.bibx28], and GARP (globally optimized alternating-phase rectangular pulses, [Bibr bib1.bibx29]). Typically, the pulses are constant in amplitude but may differ in duration. By design, the integral of the rf field $\int \omega_{1x}dt$ averages to zero over one cycle. Consequently, the contribution of $\omega_{1x}$ vanishes after each full cycle if Eq. ([Disp-formula Ch1.E3]) governs the evolution during all pulses in the cycle. In Fig. [Fig Ch1.F1]a–c, the validity of this approximation is tested for DIPSI-2 by comparing the trajectories predicted by Eq. ([Disp-formula Ch1.E3]) with exact numerical simulations as described in [Bibr bib1.bibx25]. When the initial magnetization is oriented far away from the x axis, the trajectories diverge at longer irradiation times $t_{p}$ of the DIPSI-2 pulse train. These differences stem from small contributions of the oscillating terms in Eq. (A5) due to the rapid switching of rf phases, which accumulate as the number of cycles increases. For shorter times $t_{p}<0.4$ s, the curves obtained by both methods agree quite well.

For a continuous unmodulated rf field, the trajectories of $m^{A}$ calculated with the two methods are indistinguishable, while for a GARP pulse train, where the phases are alternated 2.5 times more frequently than for DIPSI-2 with the same rf amplitude, the deviations are much larger (see the Supplement). Although one might be tempted to attribute this result to the higher phase-switching rate, the details of the sequence are also important. For example, the use of WALTZ-16, with a phase-switching rate that is 1.6 times more frequent than for DIPSI-2, results in a perfect match between the trajectories calculated with the two methods. The elementary block of WALTZ-16 consists of three pulses, $90{{}^{\circ }}_{x}180{{}^{\circ }}_{-x}270{{}^{\circ }}_{x}$ , i.e., a 180 ${}^{\circ }$ pulse flanked by two pulses whose sum is 360 ${}^{\circ }$ . These angles make the oscillating terms in Eq. (A5) run over exactly 1 and 2 full rotations.

Figure [Fig Ch1.F1]d shows simulations of the intermolecular transfer of phase coherence due to the DF from spins A to spins S for a gradient-selected selective TOCSY experiment [Bibr bib1.bibx7]. As in Fig. [Fig Ch1.F1]a–c, the solid lines have been obtained with the trajectory of $m^{A}$ , calculated by numerical integration of the non-linear coupled differential Eq. ([Disp-formula Ch1.E2]), as described in [Bibr bib1.bibx25], while the dashed lines have been obtained with the trajectory predicted by Eq. ([Disp-formula Ch1.E3]). For the latter simulations (see the supporting information for the code), Eq. ([Disp-formula Ch1.E3]) with $\omega_{1x}=0$ has been used to calculate the evolution of $m^{A}$ between the DIPSI-2 cycles, while, within each cycle, the trajectory was assumed to be solely determined by the rf field. The dashed lines that can be calculated very rapidly are barely distinguishable from the solid ones that require the more laborious simulations of [Bibr bib1.bibx25].

Effects of RD may mask or dampen those of the DF since the timescale in which RD occurs can be more than 1 order of magnitude shorter [Bibr bib1.bibx8]. However, in the experiments shown in this work, the former are suppressed by de-phasing the A spins with a 90 ${}^{\circ }$ pulse followed by a PFG, which allows us to focus on effects of the DF.

### 2.2 A qualitative description of the experiments

In the next section, several TOCSY-like experiments, where the DF mediates an intermolecular transfer of phase coherence, will be discussed. While the above theory will be used to simulate the transfer, the formalism developed by Warren and coworkers [Bibr bib1.bibx21] will be employed to guide the experimental design. For this, the high-temperature approximation needs to be abandoned by taking into account higher-order terms in the expansion of the density matrix:

4 $$\rho_{\mathrm{eq}}=\left(1-S_{z}\right)\prod_{i}\left(1-A_{z}^{i}\right).$$

Since intermolecular interactions between sparse spins S can be neglected, the index i runs over all abundant spins A . In the experiments explored in this work, the equilibrium density operator evolves during a preparation period – before the DIPSI-2 irradiation – under the influence of rf pulses and PFGs. In this period, the DF will be neglected either because of its brevity or because the relevant part of the density operator commutes with the effective dipolar Hamiltonian. The intermolecular dipolar interactions between the A and S spins need to be tracked. The effective intermolecular dipolar Hamiltonian during the DIPSI-2 sequence is given by [Bibr bib1.bibx19]:

5 $$H_{AS}^{\mathrm{eff}}=-A_{x}S_{x}+\left(A_{y}S_{y}+A_{z}S_{z}\right)/2.$$

The transfer induced by the DF can be evaluated by calculating the commutator of the density matrix and this Hamiltonian. The precise coefficients have been omitted in these equations since they are not needed for a mere qualitative description. Likewise, only the minimal number of spins A , which may cause an observable signal on the S spins, will be considered. In this work, it is sufficient to account for two-spin operators containing one A and one S spin since the different transfers involve only operators containing single-quantum (SQ) terms of the A spins (the experiment described in Sect. [Sec Ch1.S3.SS2] involves a DQ coherence which is a product of SQ terms of the A and S spins). For a qualitative description of the experiments, SQ operators containing only a single A spin are sufficient. For quantitative calculations, SQ operators containing multiple A spins (such as $A_{+}^{1}A_{-}^{2}A_{+}^{3}$ ) are also needed [Bibr bib1.bibx21]. The A spins are assumed to have the same spatial coordinates as the S spins. These simplifications preclude a precise description of the evolution due to the DF; neither the correct amplitude nor the angular dependence can be predicted. Nevertheless, it allowed good insight into the original experiment where a DIPSI-2 sequence was used to transfer the coherence between A and S spins and even provided a close estimate of the ratio of the initial rates of transfer into the different coherence orders [Bibr bib1.bibx25].

## 3 Experiments

In [Bibr bib1.bibx25], we demonstrated that the DF can efficiently mediate an intermolecular transfer of phase coherence during DIPSI-2 pulse trains. In anterior work, where the DF acted during free-precession delays, the change of coherence order needed to be achieved by rf pulses. On the contrary, the effective dipolar Hamiltonian during the DIPSI-2 irradiation allows for a change in coherence order by the DF. The transfer from a +1 to a -1 coherence was shown to be particularly efficient. This coherence order pathway refocuses $B_{0}$ inhomogeneities. In Sect. [Sec Ch1.S3.SS1], the original experiment is adapted to obtain high-resolution spectra in inhomogeneous fields in a HOMOGENIZED-like [Bibr bib1.bibx31] fashion. In Sect. [Sec Ch1.S3.SS2], an alternative coherence selection pathway will be investigated: the transfer mediated by the DF from an intermolecular DQ coherence to z magnetization. When multiple PFGs in different directions combined with several rf pulses are applied, the modulation pattern of the magnetization can become more complex. In Sect. [Sec Ch1.S3.SS3], the influence of this kind of modulation on the intermolecular transfer will be investigated.

**Figure 2 Ch1.F2:**
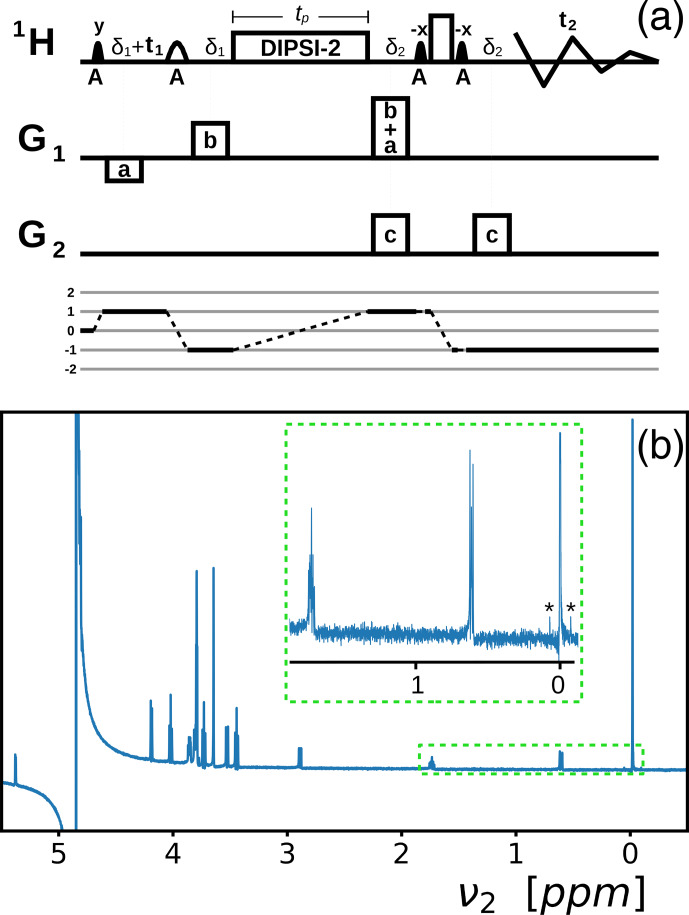
**(a)** Variant of the selective TOCSY pulse sequence that facilitates the transfer of phase coherence by the DF [Bibr bib1.bibx25] adapted to record broadband in-phase high-resolution spectra in inhomogeneous fields with solvent suppression. Narrow, filled and wide, open shapes stand for 90 and 180 ${}^{\circ }$ rf pulses, respectively. Low-amplitude pulses are selectively applied to the abundant A spins, as indicated below the pulses. The DIPSI-2 pulse train and the rectangular high-amplitude pulse are broadband. All pulses are applied along the x axis, unless specified otherwise. $G_{1}$ , $G_{2}$ , and $G_{3}$ indicate three orthogonal PFG directions, not necessarily x , y , or z . The delays $\delta_{i}$ accommodate the lengths of the PFGs (in this work, all PFGs had equal lengths). $t_{p}$ is the duration of the DIPSI-2 pulse train, and $t_{1}$ and $t_{2}$ are the indirect and direct evolution times. The coherence of the A spins is modulated by the two PFGs $G_{a}$ and $G_{b}$ . The amplitude of $G_{a}$ must be large enough to quench the effects of RD but must also be as low as possible to diminish losses due to diffusion. The area of $G_{a+b}$ must be equal to the sum of the areas of $G_{a}$ and $G_{b}$ ; the signs of the PFGs are indicated by positive or negative rectangles. In general, a PFG marked pa+qb means a PFG that is equal to the vector addition p times $G_{a}$ and q times $G_{b}$ . Purging PFGs $G_{c}$ help to suppress the signal of the A spins. The coherence selection pathway is plotted below the sequence. **(b)** A 1D spectrum recorded with $t_{1}=0$ and a DIPSI-2 irradiation time of 200 ms in a homogeneous $B_{0}$ field. The signals below 3 ppm belong to DSS (0.5 mM); among them, at 0 ppm, are the nine methyl protons (the ${}^{13}$ C satellites are marked with asterisks). The dispersive peak at 4.85 ppm stems from leftover H ${}_{2}$ O. All other signals come from sucrose (2 mM). In the supporting information, the assignments are given.

**Figure 3 Ch1.F3:**
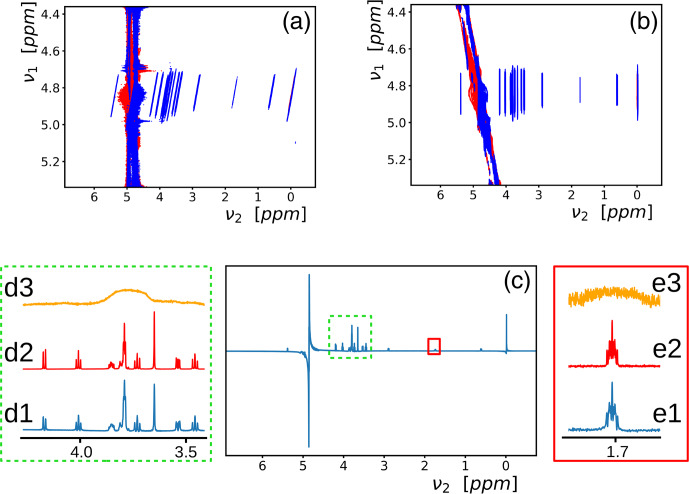
**(a)** A 2D spectrum recorded with the pulse sequence of Fig. [Fig Ch1.F2]a under the same conditions as the 1D spectrum of Fig. [Fig Ch1.F2]b, except that the $B_{0}$ field was purposely rendered inhomogeneous (line widths of about 225 Hz). **(b)** The 2D spectrum of **(a)** has been tilted, and the sliding window function of Eq. ([Disp-formula Ch1.E6]) has been applied. **(c)** The sum of the central 112 rows of **(b)**. **(d1, e1)** Two regions of the spectrum **(c)** have been enlarged. **(d2, e2)** The corresponding regions of a pulse-acquire spectrum, preceded by saturation of the solvent signal, in a homogeneous $B_{0}$ field. **(d3, e3)** The same regions from the Fourier transform of the first free induction decay (corresponding to $t_{1}=0$ ) of the experiment.

**Figure 4 Ch1.F4:**
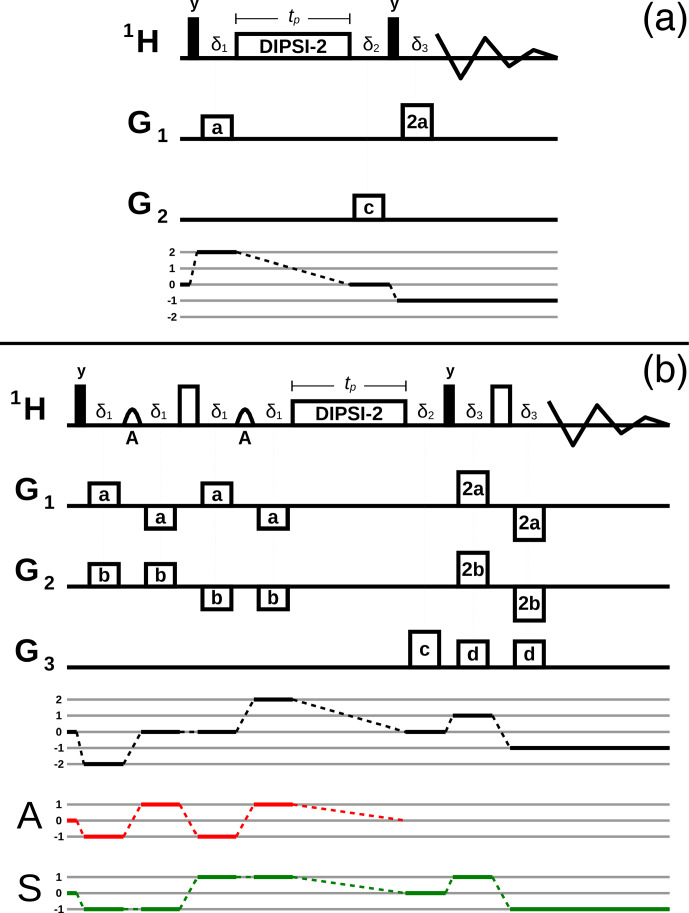
Pulse sequences where the DF mediates the transfer from a DQ coherence involving the abundant spins A and the sparse spins S to a longitudinal magnetization during the DIPSI-2 pulse train. See the caption of Fig. [Fig Ch1.F2] for an explanation of the different rf pulses, delays, and PFGs. The coherence selection pathways are plotted below the sequences. **(a)** Non-selective experiment where both the solvent spins A and solute spins S are affected by $G_{a}$ . After a purging PFG $G_{c}$ and a 90 ${}^{\circ }$ rf pulse, a PFG with twice the area of $G_{a}$ is necessary to observe the signal of the S spins. **(b)** Same type of transfer as **(a)**, with separate gradient labeling of spins A by $4\;G_{a}$ and of spins S by $4\;G_{b}$ . The signal of the S spins needs to be recovered by the sum of both PFGs. The two PFGs $G_{d}$ , while not strictly necessary, improve the solvent suppression. On the bottom, the coherence selection pathways of the A (red) and S (green) spins are also displayed separately.

### 3.1 High-resolution spectra in inhomogeneous fields

Figure [Fig Ch1.F2]a depicts an adaptation of the selective TOCSY pulse sequence [Bibr bib1.bibx25] with DIPSI-2 rf irradiation used to transfer the phase coherence from the abundant solvent spins A to the solute spins S , which includes (1) a WATERGATE spin echo [Bibr bib1.bibx26] to refocus the chemical shift evolution after the DIPSI-2 irradiation and to achieve solvent suppression and (2) an indirect evolution period before the TOCSY pulse train to record high-resolution spectra in inhomogeneous $B_{0}$ fields, similarly to experiments by [Bibr bib1.bibx16], which used the DF combined with spin echo correlation spectroscopy (SECSY, [Bibr bib1.bibx23]). In Fig. [Fig Ch1.F2]b, a 1D spectrum is obtained with $t_{1}=0$ in a homogeneous $B_{0}$ field of 2 mM sucrose and 0.5 mM sodium 3-(trimethylsilyl)propane-1-sulfonate (DSS) in a 90 % / 10 % mixture of H ${}_{2}$ O / D ${}_{2}$ O (the same sample is used throughout this work). This sample is a standard to test water suppression, with a well-dispersed distribution of resonances between 0 and 6 ppm. The inset, which shows part of the spectrum, highlights the small phase distortions at long DIPSI-2 irradiation times ( > 100 ms), which cannot be simultaneously corrected for all resonances by a linear phase correction. The ${}^{13}$ C satellites on both sides of the methyl protons (marked with asterisks) correspond to a concentration of 22.5 µ M.

The results of a 2D experiment in an inhomogeneous $B_{0}$ field are shown in Fig. [Fig Ch1.F3]a (the line width at half height measured on the water resonance was about 225 Hz). The coherence selection pathway below the sequence in Fig. [Fig Ch1.F2] shows that $B_{0}$ inhomogeneities that have evolved in $t_{1}$ should be refocused during the direct dimension at the time $t_{2}=t_{1}$ , which leads to the skewed line shapes. No corrections or special processing protocols were applied to remove phase twists in the spectrum. The absence of those are due to constructive interference of neighboring peaks (Sect. 6.5.2 of [Bibr bib1.bibx13]). Moreover, even if present, phase twists hardly perturb the results (see supporting information). In the indirect $t_{1}$ dimension, the spectrum needs to cover the inhomogeneously broadened line shape (here, 1 ppm was used). On the right (Fig. [Fig Ch1.F3]b), a sliding window function was applied in the direct $t_{2}$ dimension, and subsequently, the spectrum was sheared so that the elongated ridges appear perpendicular to the $\nu_{2}$ axis. The window function $W_{i}$ consisted of zeroes for all time points, except for a narrow range of points k={-d+o,d+o} where the intensities were multiplied by

6 $$W_{k}=1-{\mathrm{sin}}^{2n}\{\pi (k-bw_{2}t_{1})/(2d)\},$$

where $bw_{2}$ is the bandwidth in the direct dimension (the inverse of the time increment), and the offset o is the integer nearest to $bw_{2}t_{1}$ . The higher the integer value n , the closer it is to a rectangular profile. The broader the inhomogeneous line, the sharper the echo and, consequently, the smaller the range 2d . This function is identical to the amplitude modulation of wide-band, uniform-rate, and smooth-truncation pulses (WURST; [Bibr bib1.bibx20]). The variables d=300 and n=1 were optimized empirically (the value of n only slightly affects the result). Application of this window function resembles chunk selection in pure shift NMR [Bibr bib1.bibx34].

The 1D spectrum in Fig. [Fig Ch1.F3]c corresponds to the sum of the middle 112 rows (of a total of 512) of the spectrum of Fig. [Fig Ch1.F3]b. On the side panels, parts of this spectrum (Fig. [Fig Ch1.F3]d1 and e1, corresponding to a very crowded region and to a weak and complex multiplet) are compared with the results of a pulse-acquire experiment preceded by saturation of the solvent signal in a homogeneous $B_{0}$ field (Fig. [Fig Ch1.F3]d2 and e2). Fig. [Fig Ch1.F3]d3 and e3 show parts of the Fourier transform of the first increment ( $t_{1}=0$ ). The enlargements of Fig. [Fig Ch1.F3]d1 and e1 closely resemble those of Fig. [Fig Ch1.F3]d2 and e2. In the lower spectra, slight distortions due to scalar-coupling evolution during the WATERGATE spin echo are visible, and the relative peak intensities do not exactly match those in the center spectra.

The experiment presented in this section is capable of delivering broadband, in-phase, well-resolved spectra with only small distortions. It shares these characteristics with the experiment of [Bibr bib1.bibx14], which adds an adiabatic spin-lock to the method of [Bibr bib1.bibx16] in order to avoid scalar-coupling evolution during the transfer by the DF but keeps the same effective dipolar Hamiltonian as the one during free precession and achieves the changes in coherence order solely by rf pulses. Since the effective Hamiltonian is scaled during the DIPSI-2 pulse train, the transfer is slower compared to sequences which rely on prolonged free-precession delays. On the other hand, the entire (phase-modulated) magnetization of the solvent contributes to the transfer. If the solute relaxation rates are higher than the ones of the solvent (as is often the case), the new sequence should be advantaged since the losses depend on a mix of longitudinal and transverse relaxation rates. Moreover, contributions from conformational exchange to the relaxation will be attenuated. In vivo, its use may be limited due to the prolonged duration of rf irradiation.

### 3.2 Double-quantum transfer

In the original CRAZED sequence (COSY revamped with asymmetric z -gradient echo detection) of [Bibr bib1.bibx32], intermolecular DQ coherences are converted to SQ ones by a 90 ${}^{\circ }$ pulse. The DF then converts these multiple-spin SQ coherences to observable one-spin SQ coherences. The appropriate coherence order pathway needs to be selected by a judicious choice of PFGs, in this case a 1:2 ratio of the areas of the PFGs before and after the 90 ${}^{\circ }$ mixing pulse. This rf pulse is essential because the dipolar Hamiltonian does not allow for a change in coherence order. As seen in the previous section, this constraint does not apply to the effective Hamiltonian of Eq. ([Disp-formula Ch1.E5]). Hence, it may be interesting to explore the effect of the DF on intermolecular DQ coherences during DIPSI-2 irradiation.

After the first 90 ${}^{\circ }$ pulse of the sequence in Fig. [Fig Ch1.F4]a, the magnetization of the A and the S spins is de-phased by a PFG $G_{a}$ . The commutator of $H_{AS}^{\mathrm{eff}}$ of Eq. ([Disp-formula Ch1.E5]) with the lowest-order term in the expansion of the density operator, which contains a product of S and A spin operators, results in the following:

7 $$\begin{array}{rl} & -i[H_{AS}^{\mathrm{eff}},\left(c_{a}S_{x}+s_{a}S_{y}\right)\left(c_{a}A_{x}+s_{a}A_{y}\right)]\\ & =-(3/16)s_{2a}\left(S_{z}+A_{z}\right), \end{array}$$

where $c_{a}$ and $s_{a}$ stand for $\mathrm{cos}(\tau_{a}\gamma G_{a}\cdot r)$ and $\mathrm{sin}(\tau_{a}\gamma G_{a}\cdot r)$ , with $\tau_{a}$ being the effective duration of $G_{a}$ and r being the spatial position. In general, for arbitrary numbers p and q , $c_{pa+qb}$ stands for $\mathrm{cos}(p\tau_{a}\gamma G_{a}\cdot r+q\tau_{b}\gamma G_{b}\cdot r)$ . Thus, the DIPSI-2 irradiation leads to a transfer of a DQ coherence involving the A and S spins to longitudinal magnetization. A purge gradient followed by a 90 ${}^{\circ }$ rf pulse and a final PFG $+2G_{a}$ or $-2G_{a}$ ( $pG_{a}$ stands for a PFG along the direction of $G_{a}$ with an area p times as large) allows one to detect the longitudinal term of the sparse S spins. A phase difference ϕ due to chemical shift evolution causes a phase shift ϕ in the signal and additional longitudinal components which are not modulated by the gradient.

In Fig. [Fig Ch1.F5]a, the intensity, $m_{xy}=\sqrt{m_{x}^{2}+m_{y}^{2}}$ , of the methyl proton resonance of DSS is plotted as a function of the orientation angle $\Theta_{G_{a}}$ of the PFG $G_{a}$ for a DIPSI-2 irradiation time of about 100 ms. The line that goes through the points corresponds to a function $m_{xy}^{0}(3{\mathrm{cos}}^{2}\Theta_{G_{a}}-1)/2$ , where $m_{xy}^{0}$ is the intensity recorded with the PFG oriented parallel to $B_{0}$ . The line crosses zero at the so-called magic angle Θ=54.74 ${}^{\circ }$ . Although, by definition, all intensities are positive, for clarity, the intensities of signals that point in opposite directions when phased identically are plotted with opposite signs. The theoretical curve should match the experimental points only at short irradiation times (i.e., in the linear regime of the buildup).

In Fig. [Fig Ch1.F5]b, the buildup of intensities is plotted as a function of the irradiation time $t_{p}$ for orientations that are parallel ( $\Theta_{G_{a}}=0{}^{\circ }$ , blue squares) and perpendicular ( $\Theta_{G_{a}}=90{}^{\circ }$ , blue circles) with respect to $B_{0}$ . The dot-dashed blue lines are simulations of these experiments using the approximate solution of the non-linear Bloch Eq. ([Disp-formula Ch1.E2]) for the evolution of $m^{A}$ . The intensities for $\Theta_{G_{a}}=90{}^{\circ }$ at longer irradiation times exceed half of those for $\Theta_{G_{a}}=0{}^{\circ }$ . This is because, when the magnitude of $\omega_{d}$ is divided by a factor of 2, the transfer is not half as efficient but rather twice as slow (of course, a slower transfer renders the experiment more sensitive to losses due to relaxation and diffusion). The red crosses are the intensities obtained with the experiment described in [Bibr bib1.bibx25] (i.e., the experiment of Fig. [Fig Ch1.F2]a but without refocusing pulses, solvent suppression, and an indirect evolution period) for $\Theta_{G}=0{}^{\circ }$ and a coherence pathway selection +1→-1 . For clarity, the latter intensities have been divided by 2 since the (simplified) commutator formalism predicts that the initial slopes should differ by a factor 2. This factor applies only for the initial slopes. Neglecting relaxation and diffusion, the transfer reaches a theoretical maximum of $m_{xy}^{S}=0.88$ at $t_{p}\approx 450$ ms for the experiment of Fig. [Fig Ch1.F2] and of $m_{xy}^{S}=0.32$ at $t_{p}\approx 320$ ms for the experiments of Fig. [Fig Ch1.F4].

**Figure 5 Ch1.F5:**
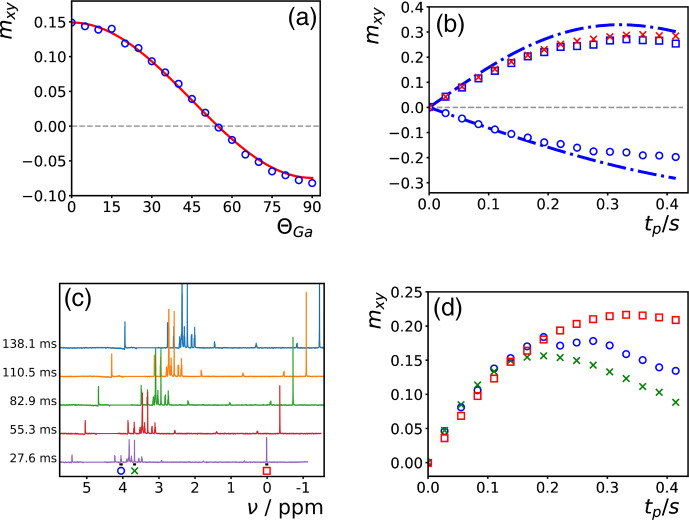
**(a)** Signal intensities of the methyl protons of DSS obtained with the sequence of Fig. [Fig Ch1.F4]a with a DIPSI-2 irradiation time of 100 ms. The orientation of $G_{a}$ was varied, while its amplitude was kept constant. While $m_{xy}$ is always positive, it has been multiplied by -1 for signals that were opposite with respect to the first one. The red line is a function proportional to $3{\mathrm{cos}}^{2}\Theta_{\mathrm{Ga}}-1$ that goes through the first point. **(b)** At the angles $\Theta_{\mathrm{Ga}}=0{}^{\circ }$ (blue squares) and $\Theta_{\mathrm{Ga}}=90{}^{\circ }$ (blue circles), the evolution of the signal has been recorded as a function of DIPSI-2 irradiation time $t_{p}$ . The dot-dashed lines are simulations as explained in the main text (without taking into account relaxation or translational diffusion). The red crosses are intensities, scaled down by a factor 2, obtained with the experiment described by [Bibr bib1.bibx25]. **(c)** Spectra of a solution of 2 mM sucrose and 0.5 mM DSS in H ${}_{2}$ O / D ${}_{2}$ O (90 % / 10 %) recorded with the experiment of Fig. [Fig Ch1.F4]b for several irradiation times $t_{p}$ indicated on the left side of the spectra. The baseline of each spectrum has been corrected separately for the regions on the left and right of the solvent signal, which has been digitally removed for clarity. **(d)** The buildup curves of three selected resonances (see supporting information for the assignments) marked at the bottom of **(c)**.

In the sequence of Fig. [Fig Ch1.F4]b, the solvent and solute spins are labeled by different gradients, and the chemical shifts are refocused. The A spins are de-phased by $4G_{a}$ and the S spins by $4G_{b}$ . During the DIPSI-2 irradiation, the DF induces the following transfer:

8 $$\begin{array}{rl} & -i[H_{AS}^{\mathrm{eff}},\left(c_{4b}S_{x}-s_{4b}S_{y}\right)\left(c_{4a}A_{x}-s_{4a}A_{y}\right)]\\ & =(3/16)s_{4a+4b}S_{z}-(1/16)s_{4a-4b}S_{z}+\ldots \end{array}$$

As in the previous sequence, only single-spin SQ terms of the A spins suffice for a qualitative description. The first term on the right can be recovered after a 90 ${}^{\circ }$ pulse by the sum of the two PFGs, $\pm (4G_{a}+4G_{b})$ . The solvent senses only $G_{a}$ before the DIPSI-2 irradiation so that no additional measures need to be taken to suppress its signal after the DIPSI-2 irradiation, and, consequently, the echo before signal detection is shorter than the one in Fig. [Fig Ch1.F2]. The characteristic frequency $\omega_{d}$ of the DF depends only on the orientation of $G_{a}$ .

In Fig. [Fig Ch1.F5]c, spectra obtained with $G_{a}$ along the z axis and with $G_{b}$ along the x axis for several DIPSI-2 irradiation times are displayed. The buildup curves of three resonances (the methyl resonance of DSS and two sucrose resonances) are plotted in Fig. [Fig Ch1.F5]d. The buildup curves are very similar at short times and start to diverge at longer times, probably due to differences in relaxation rates.

**Figure 6 Ch1.F6:**
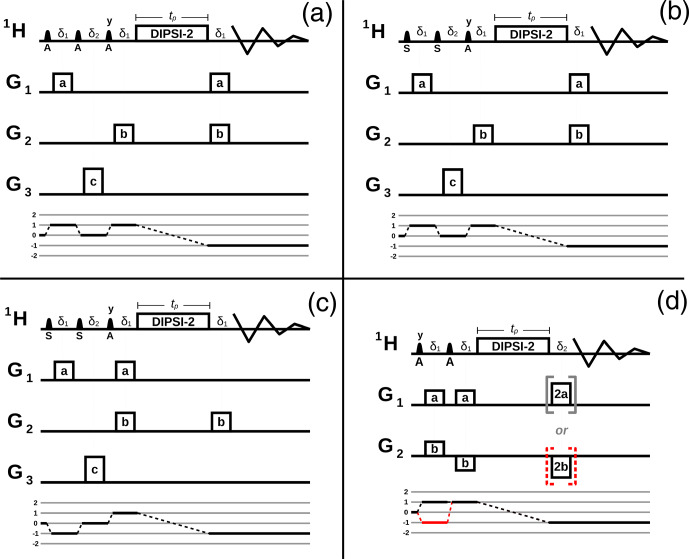
Variants of pulse sequences that record the transfer of phase coherence order from +1 to -1 by the DF. See the caption of Fig. [Fig Ch1.F2] for an explanation of the different rf pulses, delays, and PFGs. **(a)** A PFG $G_{a}$ sandwiched between two selective 90 ${}^{\circ }$ pulses modulates the amplitude of the A magnetization. After a purging PFG $G_{c}$ and a 90 ${}^{\circ }$ pulse, $G_{b}$ modulates the phase of the A spins. Following the DIPSI-2 irradiation, the newly created coherence on the S spins can be recovered by the two PFGs $G_{a}$ and $G_{b}$ . **(b)** Similar to **(a)**, except that the first two 90 ${}^{\circ }$ pulses are selectively applied on the S spins. **(c)** Similar to **(b)**, except that the gradient $G_{a}$ has been moved in front of the DIPSI-2 irradiation. The coherence selection pathway is different compared to **(a)** and **(b)**. **(d)** The combination of PFGs and rf pulses before the DIPSI-2 pulse train creates two DFs due to a modulation of the magnetization of A in different directions, both of them simultaneously mediating a transfer of phase coherence. Either the modulation due to $G_{a}$ or the one due to $G_{b}$ is recovered. When the gradient $2G_{a}$ is used, the selected coherence order after the first rf pulse is +1 , while for $-2G_{b}$ , it is -1 (red line).

### 3.3 Mixed modulations

When applying multiple rf pulses interleaved with PFGs in different directions, the modulation of the magnetization can become rapidly very convoluted. Often, PFGs serve as purging gradients, and the parts of the density operator that are de-phased by these PFGs can be discarded. However, magnetization which is modulated in complex patterns can also be implicated in a transfer of phase coherence by the DF, although the conditions for the validity of the theory described in Sect. [Sec Ch1.S2.SS1] may not strictly apply anymore. In this section, this type of more intricate modulations will be investigated. Several schemes will be presented where the density operator is prepared in different ways and where the transfer from a +1 to a -1 coherence order is recorded.

At the start of the sequence of Fig. [Fig Ch1.F6]a, a PFG $G_{a}$ sandwiched between two selective 90 ${}^{\circ }$ pulses modulates the amplitude of the magnetization of the abundant A spins. It is followed by a purging PFG and another selective pulse and PFG $G_{b}$ before DIPSI-2 irradiation. Neglecting the parts of the density operator which are de-phased by $G_{c}$ , the transfer due to the DF that occurs during the DIPSI-2 irradiation can be described as follows:

9 $$\begin{array}{rl} & -i[H_{AS}^{eff},S_{z}\left(c_{a}c_{b}A_{x}+c_{a}s_{b}A_{y}\right)]\\ & =(2/16)c_{a}s_{b}S_{x}+(4/16)c_{a}c_{b}S_{y}+\ldots \end{array}$$

Using the relations $c_{a}c_{b}=(c_{b+a}+c_{b-a})/2$ and $c_{a}s_{b}=(s_{b+a}+s_{b-a})/2$ , the DF can be decomposed into two fields, one originating from the half of the magnetization of the A spins that is de-phased by $G_{b}-G_{a}$ and the other from the half that is de-phased by $G_{b}+G_{a}$ . If the gradients $G_{b}$ and $G_{a}$ are oriented either parallel or perpendicular to $B_{0}$ , both fields are characterized by the same value of $\omega_{d}$ . With the first two pulses applied selectively to the S instead of to the A spins, shown in Fig. [Fig Ch1.F6]b, the operator analysis remains the same so that Eq. ([Disp-formula Ch1.E9]) also describes the transfer in this case.

The experiments of Fig. [Fig Ch1.F6]a and b were performed with $G_{b}$ along z and with $G_{a}$ along x and a DIPSI-2 irradiation time of 100 ms. The areas of the PFGs have been varied to change the angle $\Theta_{G_{a}+G_{b}}$ of the vector addition of $G_{a}$ and $G_{b}$ with respect to $B_{0}$ while keeping the amplitude constant. The transfer efficiency is plotted as a function of $\Theta_{G_{a}+G_{b}}$ in Fig. [Fig Ch1.F7]a and b for the experiments of Fig. [Fig Ch1.F6]a and b, respectively. Only in the first experiment where all selective pulses are applied to H ${}_{2}$ O is the typical $\left(3{\mathrm{cos}}^{2}\Theta -1\right)$ dependence observed. The direction of the spatial modulation of the A spins is important; this does not change with $\Theta_{G_{a}+G_{b}}$ in the experiment of Fig. [Fig Ch1.F6]b since the first two pulses are applied on the S spins. While, for intramolecular two-spin operators, it does not matter how the amplitude or phase modulation has been created, for intermolecular two-spin operators, Eq. ([Disp-formula Ch1.E9]) does not provide the full picture. For a spin S at a given position, one has to consider the dipolar interactions with all spins A [Bibr bib1.bibx21], whose spatial modulation is different for the two experiments. The $\left(3{\mathrm{cos}}^{2}\Theta_{G_{a}+G_{b}}-1\right)$ dependence, which is absent in the experiment of Fig. [Fig Ch1.F6]b, is restored in the sequence of Fig. [Fig Ch1.F6]c, where the second gradient $G_{a}$ is put before the DIPSI-2 block instead of behind, as shown in Fig. [Fig Ch1.F7]c.

For the experiment of Fig. [Fig Ch1.F6]a, Fig. [Fig Ch1.F7]d shows the transfer as a function of the DIPSI-2 irradiation time at angles $\Theta_{G_{a}+G_{b}}=15{}^{\circ }$ (blue circles) and $\Theta_{G_{a}+G_{b}}=80{}^{\circ }$ (green triangles). The dot-dashed curves are simulations that assume $\omega_{d}$ is constant throughout the irradiation time, which may not be entirely correct since the spatial pattern of the modulations of the magnetization of A will change over time (although the modulations will remain in the plane spanned by the vectors in the directions of $G_{b}-G_{a}$ and $G_{b}+G_{a}$ ). The red crosses correspond to the experiment of Fig. [Fig Ch1.F6]c, with $\Theta_{G_{a}+G_{b}}=15{}^{\circ }$ . The buildup is almost indistinguishable from the one in blue circles. The simulated curves for the latter experiment (here, $\omega_{d}$ is constant since the magnetization of A is modulated in one direction only) are almost identical to the previous ones, indicating that the assumption of a constant $\omega_{d}$ , as used for the simulations of experiments of Fig. [Fig Ch1.F6]a, is a reasonable approximation for these DIPSI-2 irradiation times.

**Figure 7 Ch1.F7:**
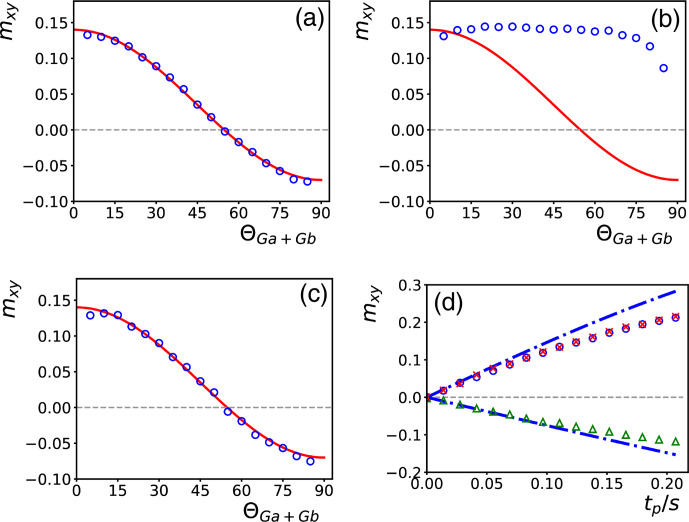
**(a–c)** Signal intensities of the methyl protons of DSS obtained with the pulse sequences of Fig. [Fig Ch1.F6]a–c. A DIPSI-2 irradiation time of about 100 ms has been used. The angle $\Theta_{G_{a}+G_{b}}$ , i.e., the angle between the vector addition of PFGs $G_{a}$ and $G_{b}$ and $B_{0}$ , has been varied between 5 and 85 ${}^{\circ }$ while keeping the amplitude constant. The red lines, which correspond to a function $0.14\times (3{\mathrm{cos}}^{2}\Theta_{G_{a}+G_{b}}-1)/2$ , match the experimental points in **(a)** and **(c)**. **(d)** Signal intensities as a function of DIPSI-2 irradiation time $t_{p}$ , recorded under the same conditions as in **(a)**, with $\Theta_{G_{a}+G_{b}}=15{}^{\circ }$ (blue circles) and 80 ${}^{\circ }$ (green triangles), and as in **(c)**, with $\Theta_{G_{a}+G_{b}}=15{}^{\circ }$ (red crosses). The dot-dashed curves are simulations, as explained in the main text.

In the experiments above, the modulation of the magnetization was decomposed into two directions, not necessarily orthogonal, each at the same angle with respect to $B_{0}$ . This is different in the following experiment. Just before the DIPSI-2 irradiation in the pulse sequence of Fig. [Fig Ch1.F6]d, the two-spin $S_{z}A_{z}$ term of $\rho_{\mathrm{eq}}$ in Eq. ([Disp-formula Ch1.E4]) has evolved into the following:

10 $$\begin{array}{rl} \rho & =S_{z}\{A_{x}c_{a+b}c_{a-b}+A_{y}c_{a+b}s_{a-b}\}\\ & =S_{z}\{A_{x}\left(c_{2a}+c_{2b}\right)/2+A_{y}\left(s_{2a}-s_{2b}\right)/2\}. \end{array}$$

A term containing $S_{z}A_{z}$ (created by the second selective pulse) has been left out since it commutes with the effective dipolar Hamiltonian of Eq. ([Disp-formula Ch1.E5]) and will not lead to an observable signal. Equation ([Disp-formula Ch1.E10]) corresponds to a situation where half of the magnetization is modulated in the direction of $G_{a}$ , and the other half is modulated in the direction of $G_{b}$ . Experiments were performed with $G_{a}$ being parallel and $G_{b}$ being perpendicular to $B_{0}$ , with different irradiation times. The blue circles in Fig. [Fig Ch1.F8] correspond to the signal intensities of the methyl protons of DSS while using a PFG $2G_{a}$ before detection. The green squares correspond to intensities recorded using a PFG $-2G_{b}$ . The signal intensities represented by the red crosses were recorded under the same circumstances as the second set of experiments (i.e., with $-2G_{b}$ before acquisition), except that $G_{a}$ was perpendicular to both $G_{b}$ and $B_{0}$ . The intensities of the latter experiments at longer times are slightly higher than the intensities of the green squares. This is likely caused by the larger perturbation of the longitudinal magnetization of the S nuclei that occurs when one of the modulations is parallel to the $B_{0}$ field.

When the magnetization is modulated in only one dimension, the strength of the DF scales with the local value of $m^{A}$ . This is not true anymore with the last scheme of this section since the DF can be decomposed into two different parts, each with its own characteristic value of $\omega_{d}$ . Remarkably, for a given position in the sample, the magnetization of the solute $m^{S}$ may be affected by the DF; albeit, the solvent magnetization vanishes ( $m^{A}=0$ ) at that position. Another counterintuitive feature is that, even if the vector addition of the PFGs after the first rf pulse and the vector addition of the PFGs after the second rf pulse both point along the magic angle with respect to $B_{0}$ , it is still possible to observe a transfer of phase coherence. Hence, application of PFGs oriented along the magic angle does not always suffice to suppress the effects of the DF.

**Figure 8 Ch1.F8:**
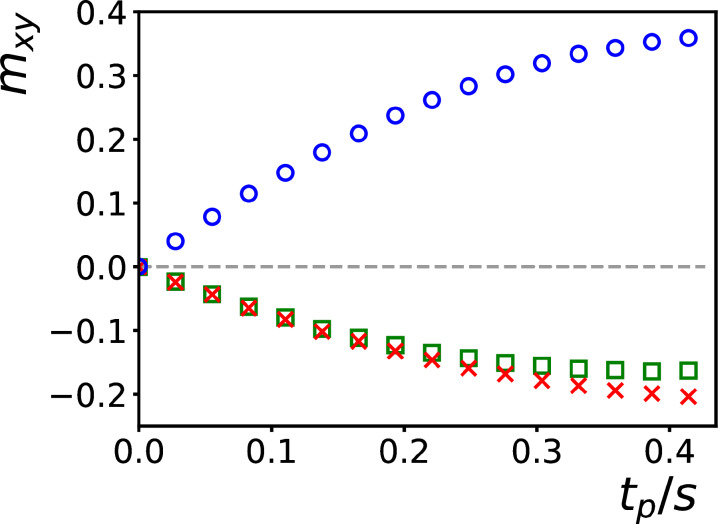
Buildup of the intensities of the methyl protons of DSS obtained with the experiment of Fig. [Fig Ch1.F6]d at different DIPSI-2 irradiation times $t_{p}$ . $G_{a}$ was parallel to $B_{0}$ , and $G_{b}$ was perpendicular. The blue circles were recorded with $2G_{a}$ as the last gradient before acquisition, and the green squares were recorded with $-2G_{b}$ as the last gradient. The red crosses were recorded under the same conditions as the green squares, except that orientation of $G_{a}$ was perpendicular to $B_{0}$ and $G_{b}$ .

## 4 Experimental parameters

All experiments were acquired in a $B_{0}$ field of 18.8 T (800 MHz proton frequency) at 290 K, with a probe equipped with coils to generate PFGs along three orthogonal axes. The rf amplitude $\omega_{1}/2\pi$ during the DIPSI-2 pulse train was 8.33 kHz. The selective pulses on either the solvent or the methyl protons of DSS had Gaussian shapes and a length of 5 ms (for both the 90 and 180 ${}^{\circ }$ pulses), except the two 90 ${}^{\circ }$ pulses in the WATERGATE scheme of Fig. [Fig Ch1.F2], which had a sinc profile and a duration of 2 ms. The shaped pulses that were applied on the equilibrium magnetization of the abundant spins A were calibrated separately to compensate for the effects of RD. All PFGs had smoothed square profiles and durations of 1 ms. $G_{1}$ , $G_{2}$ , and $G_{3}$ indicate orthogonal gradient channels. The amplitudes of the PFGs were $G_{a}=1.6$ , $G_{b}=6.2$ , $G_{a+b}=7.8$ , and $G_{c}=32$ G cm ${}^{-1}$ for the experiments of Figs. [Fig Ch1.F2] and [Fig Ch1.F3]; $G_{a}=7.8$ and $G_{c}=27$ G cm ${}^{-1}$ for Fig. [Fig Ch1.F5]a and b; $G_{a}=1.95$ , $G_{b}=1.95$ , $G_{c}=27$ , and $G_{d}=10$ G cm ${}^{-1}$ for Fig. [Fig Ch1.F5]c and d; $\|G_{a}+G_{b}\|=7.8$ and $G_{c}=32$ G cm ${}^{-1}$ for Fig. [Fig Ch1.F7]; and $G_{a}=3.9$ and $G_{b}=3.9$ G cm ${}^{-1}$ for Fig. [Fig Ch1.F8]. For all experiments, 8192 complex points were acquired with a bandwidth of 12 ppm. All signal intensities that have been quantified have been normalized either to the spectrum of the methyl protons after a selective excitation or to a broadband pulse-acquire experiment preceded by saturation of the solvent signal.

The 2D spectra of Fig. [Fig Ch1.F3] have been acquired with 256 $t_{1}$ increments, a bandwidth of 1 ppm, four scans per increment in the indirect dimension, and a repetition time of 11 s (in the supporting information, a similar experiment recorded with only one scan, a repetition time of 3 s, and a DIPSI-2 irradiation time of 100 ms is shown). For these spectra, the experimental data have been doubled in each dimension by zero padding. The spectrum of Fig. [Fig Ch1.F3]a has been obtained by a 2D Fourier transform without any apodization. For the spectrum of Fig. [Fig Ch1.F3]b, the same data have been multiplied by the window function of Eq. ([Disp-formula Ch1.E6]) and subsequently Fourier transformed along the indirect $t_{1}$ dimension. A first-order phase correction along the direct $t_{2}$ dimension, proportional to the $\nu_{1}$ position, was then applied to shear the spectrum followed by a Fourier transform along the same dimension.

## 5 Conclusions

In this work, we have investigated several aspects of the transfer of phase coherence by the dipolar field during rf irradiation sequences that have been developed for total correlation spectroscopy, in particular the DIPSI-2 pulse train. Theoretical expressions for the evolution of the solvent spins under continuous rf irradiation have been derived, which permits efficient simulations of the transfer process. A remarkable feature is that, under these conditions, the DF can cause not only a transfer but also a change in coherence order. Nevertheless, the formalism developed by Warren and coworkers [Bibr bib1.bibx21] can still be used – taking into account the effective Hamiltonian of Eq. ([Disp-formula Ch1.E5]) – to describe and design pulse sequences. An experiment for the acquisition of broadband, in-phase, high-resolution spectra in inhomogeneous fields has been presented. In this experiment, the transfer takes place from a SQ coherence of the abundant solvent spins to another SQ coherence of the sparse solute spins. Additionally, alternative coherence order pathways have been investigated: a DQ coherence involving both solvent and solute spins can be converted by the DF into longitudinal magnetization. Two pulse sequences to record this transfer have been introduced. In the first sequence, the DQ coherence is de-phased by a PFG, while the SQ coherence, which is detected, is re-phased with a PFG that is twice as strong. In the second sequence, the solvent and solute parts of the DQ coherence are de-phased by two different PFGs. The SQ coherence is now re-phased by the sum of these two PFGs. The latter sequence has the advantage of resulting in broadband, in-phase spectra without the need for additional water suppression before acquisition. In the last part, more complex modulation patterns of the magnetization have been investigated. The importance of how the magnetization of the different spins has been modulated in intermolecular multiple-spin operators has been explored with three closely resembling pulse sequences. Finally, by combining several pulsed field gradients and rf pulses, the DF has been tailored in such a manner that it can be decomposed into different components that have their own spatial modulation and, hence, can simultaneously bring about different transfer processes.

In the different sequences, DIPSI-2 pulse trains have been applied. Other TOCSY mixing sequences can be used, although one has to take into account the different effective dipolar Hamiltonians that characterize these sequences [Bibr bib1.bibx18]. These effects could be suppressed by the use of tailored mixing sequences [Bibr bib1.bibx17] or the use of PFGs along the magic angle. However, the results in Sect. [Sec Ch1.S3.SS3] caution against the latter option for sequences with many pulses and PFGs. The effects of the transfer by the scalar couplings during the TOCSY sequence have not been taken into account. This is reasonable because either an uncoupled nucleus has been probed or because the solute coherences all had the same phase or were all along the z axis before the DIPSI-2 irradiation, which minimizes the effects of the transfer by scalar couplings [Bibr bib1.bibx6]. In sequences where the chemical shifts of the solute spins evolve before the TOCSY pulse train, a more complex behavior is expected.

## Supplement

10.5194/mr-4-271-2023-supplement The supplement related to this article is available online at: https://doi.org/10.5194/mr-4-271-2023-supplement.

## Data Availability

The Python pulse program used for different simulations can be found in the Supplement.

## Appendix

The supplement related to this article is available online at: https://doi.org/10.5194/mr-4-271-2023-supplement.
