# Supplementary material for: Various facets of intermolecular transfer of phase coherence by nuclear dipolar fields
Source: Magn Reson (Gott). 2023 Dec 21;4(2):271–83. doi: 10.5194/mr-4-271-2023 (PMC10776886; doi:10.5194/mr-4-271-2023)
Supplement: The supplement related to this article is available online at: https://doi.org/10.5194/mr-4-271-2023-supplement. [file mr-4-271-supplement.pdf]

Supplement of Magn. Reson., 4, 271–283, 2023
https://doi.org/10.5194/mr-4-271-2023-supplement
© Author(s) 2023. CC BY 4.0 License.

Open Access
MAGNETIC
RESONANCE

Supplement of

Various facets of intermolecular transfer of phase coherence by nuclear dipolar fields

Philippe Pelupessy

Correspondence to: Philippe Pelupessy (philippe.pelupessy@ens.psl.eu)

The copyright of individual parts of the supplement might differ from the article licence.

Contents

Four additional figures and a simulation program.

- Figure S1) Simulations GARP 2
- Figure S2) Short experiment in inhomogeneous field 3
- Figure S3) Line shapes in 2D experiments in inhomogeneous fields 4
- Figure S4) 1D spectrum with assignments 5
- Code simulation program (parameter file) 6
- Code simulation program (main) 7-11

Figures

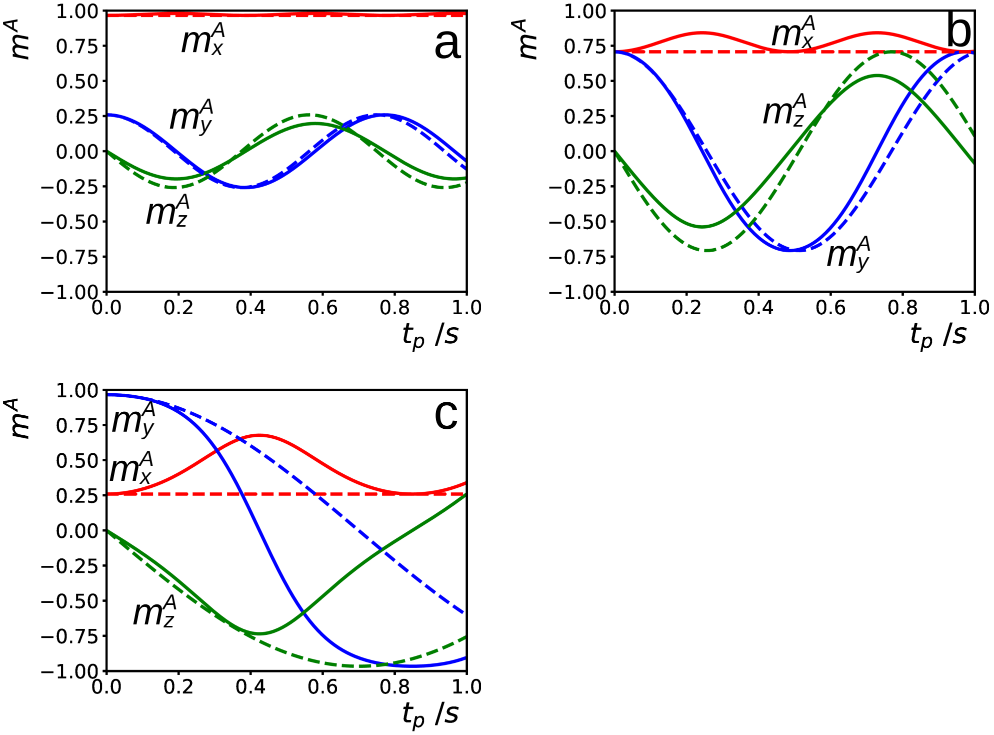

Figure S1. Same as fig. 1a-c in the main text, except that the rf pulse-train was GARP instead of DIPSI-2. All other conditions for the simulations were identical.

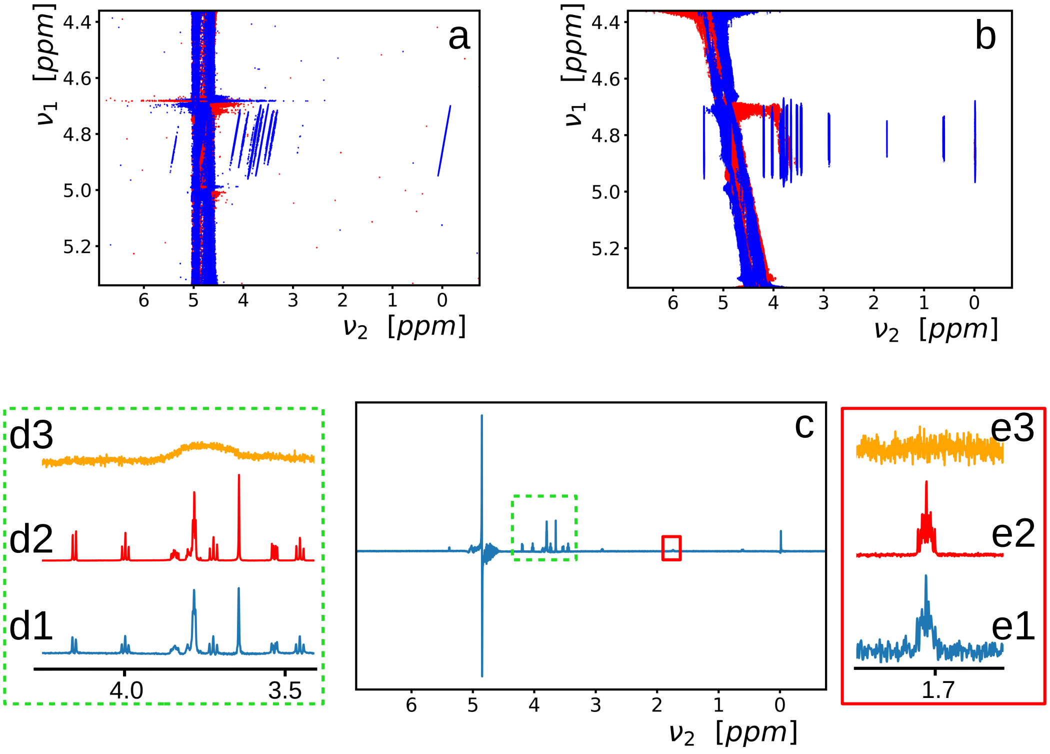

Figure S2. Results of an experiment similar to the one of fig. 3 of the main text. The sole differences were a repetition time of about 3 s instead of 11 s, a DIPSI-2 irradiation time of 100 ms instead of 200 ms, and 1 scan per increment instead of 4. This results in an experimental time of about 12 minutes instead of 3 hours.

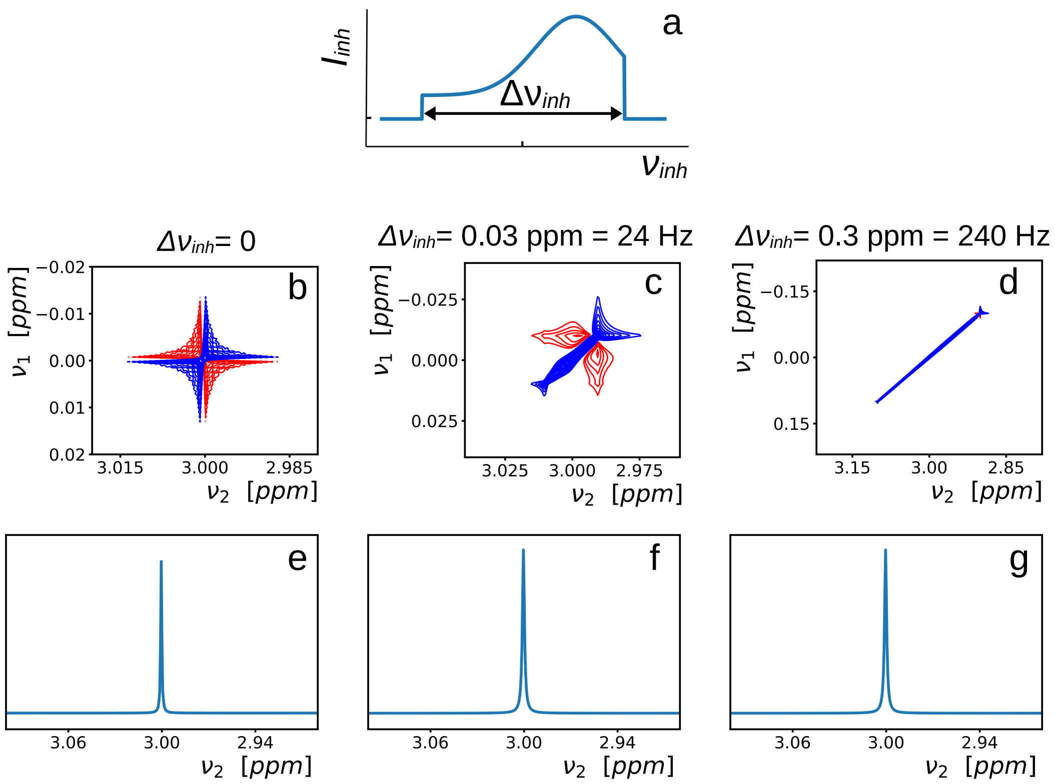

Figure S3. Calculated line shapes resulting from the experiment of fig. 2 of the main text. **(a)** The profile that characterizes the B_0 inhomogeneities, which is considered in the calculations. For a spin species S, the experiment results in:

$$sig(S) = \int cI_{inh}e^{(-i\omega_{inh}-R_2^A)t_1}e^{(i\omega_{inh}+i\omega_0^S-R_2^S)t_2}d\omega_{inh}, \tag{S1}$$

where c is a constant that depends on several factors such as the spin-density and transfer and detection efficiencies. R_2^A and R_2^S are the transverse relaxation times of the A and S spins. The signal $sig(S)$ has been calculated for proton spin S with $\omega_0^S = 3$ ppm, R_2^A = 2 s^{-1} and R_2^S= 2 s^{-1} at a field of B_0 = 18.8 T (800 MHz proton frequency). 12×1024 complex points for a bandwidth of 12 ppm were used in the direct t_2 dimension and 1024 indirect t_1 increments for a bandwidth of 1 ppm (more points have been used than in the experiments of fig. 3 in the main manuscript and fig. S2 to avoid truncation effects). In the center row the real part of the 2D Fourier transform of eq. S1 for three different scenarios is plotted: **(b)** a homogeneous B_0 field ($\Delta\nu_{inh} = 0$), **(c)** a moderately inhomogeneous field ($\Delta\nu_{inh} = 0.03$ ppm), and **(d)** a substantially inhomogeneous field ($\Delta\nu_{inh} = 0.3$ ppm). The spectra zoom in on the region of interest and the lowest contour levels are at 2% of the maximum intensity of each spectrum. Since only 1 coherence pathway is recorded, the spectrum cannot be phased to pure absorption mode, as evidenced by the severe phase twist in (b). However, due to the symmetry of these distortions, the phase twists do not appear in the sum of the rows as shown in **(e)**. The phase-twists are attenuated with increasing B_0 inhomogeneities, and are not visible in the sum, after shearing of the 2D spectra, of the rows as shown below the spectra (c) and (d) in **(f)** and **(g)**. The sum spectra in (e-g) have the same normalization factor. Thus increased inhomogeneities do not reduce the signal intensity, but, since the signal is spread out over more rows, lead to a decrease of the signal-to-noise ratio. The line-width in (e) depend on R_2^S, the line-width in (f) and (g) on the sum $R_2^A + R_2^S$.

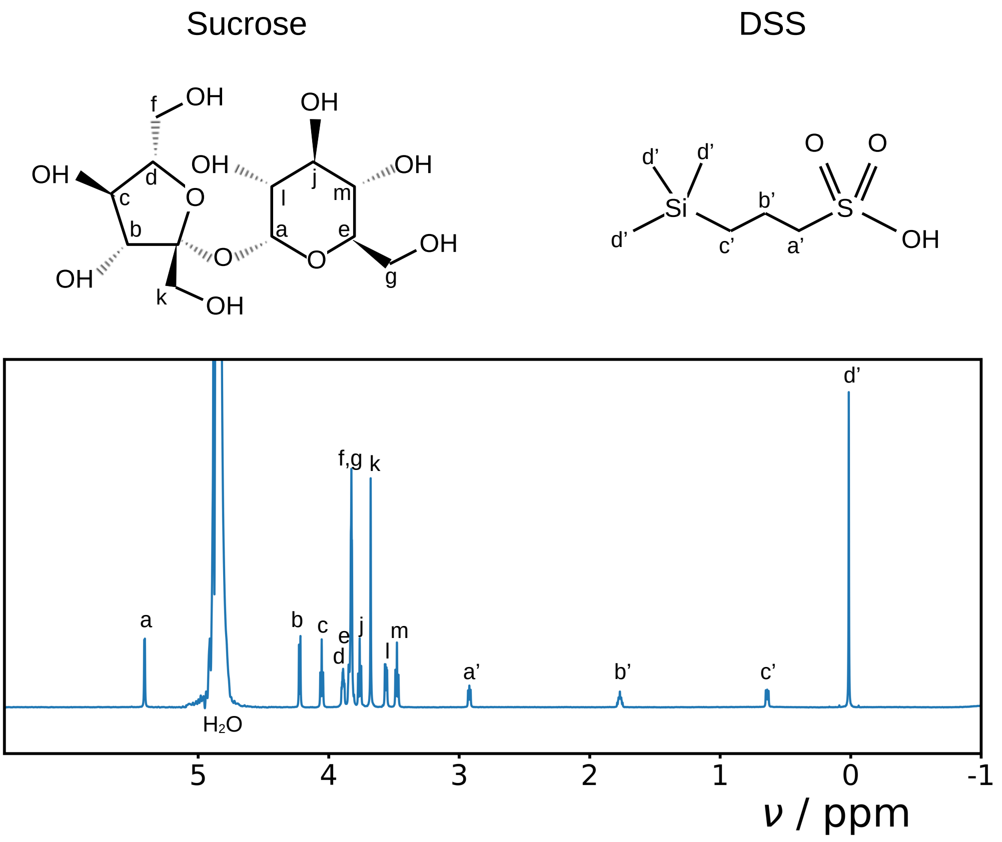

Figure S4. Spectrum obtained with a pulse-acquire experiment preceded by saturation of the solvent signal (H_2O). The sample, 0.5 mM DSS and 2.0 mM sucrose in a 90%/10% mixture of H_2O/D_2O, has been used for all experiments in this work. The assignments come from the Spectral Database for Organic Compounds, SDBSWeb, https://sdbs.db.aist.go.jp (National Institute of Advanced Industrial Science and Technology, date of access: 05-09-2023).

Program

```
'''
modified: 21-07-2023
Parameter file for simul_df_tocsy_01.py
    For fig. 1d use:
        iniDSS = "zed"
        vDF = 1.84
        order = [-1, 1]
        we look at the 'planeabs' results
    For fig. 5b use:
        iniDSS = "MH2O"
        vDF = 1.84 or -0.5*1.84
        order = [2]
        we look at the 'zabs' results
'''
B0 = -800.13        # main field (minus sign for positif gyrom.)
vDSS = -4.85        # chemical shift difference wr H2O (ppm)
vDF = 1.84          # amplitude dipolar field

mixing = "Dipsi2"   # "Dipsi2","Garp","cw" or "Waltz16"
tau90 = 30.e-6      # duration 90 pulse, defines rf power mixing
maxcyc = 120        # maximum number of mixing cycles

ngrid = 36          # number of equidistant (in angles) gridpoints for
                    # the dephased solvent magnetization (MH2O)
iniDSS = "MH2O"     # "iks", "why","zed" or "MH2O"
                    # MH2O is initially dephased in the plane, MDSS (the solute
                    # magnetization) can be along one of the axis or initially
                    # aligned with MH2O
order = [2]         # indicates the ratio beween the pfg before acquisition
                    # and the dephasing pfg
plot = True
plotopt = {'plane':False, 'planeabs':True,'z':False,'zabs':True}
                    # 'zed' plots the magnetization in the plane after:
                    #  pfg-mixing-spoiling-90-pfg*order
                    # 'plane' plots the magnetization in the plane after:
                    #  pfg-mixing-pfg*order
plotcol = ['blue','red','green','orange','cyan']
savedata = False
```

```
'''
modified: 21-07-2023
Simulation program transfer of phase coherence from abundant spins (H2O)
to solute (DSS) by dipolar field during TOCSY.
Only with mixing sequences along one axes with constant rf amplitude.
The solvent magnetization evolves as described in the main article.

Program uses numba for jit compilation. Uses para02.py file for parameters
so that numba can use cache.

The program has been run with versions:

Python     3.11.3
Numpy      1.25.1
Numba      0.57.1
Matplotlib 3.7.2

This code is provided for the purpose of checking and/or reproducing the
simulations of the main article and comes without any warranty. If you use
(part of) this code for your own work, please cite the original publication.

2023  Philippe Pelupessy
'''

# Import necessary libraries
import numpy as np
import matplotlib.pyplot as plt
from numba import njit,prange
import para02 as pa   #import parameter file
import importlib
importlib.reload(pa) #reload parameter file, needed if rerun from ipython

# Evolution of the solute magnetization
@njit(parallel=True,cache = True,fastmath=True)
def evolveDSS(MDSS,ngrid,maxcyc,MH2O,SupCyc,Angle,Phase,
              IA,sA,cA,rfA,wDSS,lA,lS,unit):
    '''Rotation around an axis during spinlock sequence along the x-axis. The
       axis of rotation for DSS is determined by the rf field and by the MH2O.
       RF power is constant but sign may alternate. On-resonance for abundant
       solvent spins, while magnetization of the off-resonant sparse spins is
       calculated. Optimized for numba just in time compilation. Gains in speed
       using fastmath=True depend strongly on computer.'''
    pi1 = np.pi/(180*unit)
```

```
    for i in prange(ngrid): #loop over all sample positions
        mxm,mym,mzm = MDSS[0,i,0],MDSS[1,i,0],MDSS[2,i,0] #initial MDSS
        c = 0
        for k in range(maxcyc):
            X,Y,Z = MH2O[0,i,k],MH2O[1,i,k],MH2O[2,i,k] #precalculated MH2O
            #at beginning of each super-cycle. Very little difference if one
            #takes average of k and k+1.
            for l in range(lS): #loop 'supercycle'
                for m in range(lA):#loop 'subcycle'
                    sig = Phase[m]*SupCyc[l] #sign rf
                    Xs,Ys,Zs = rfA*sig-0.5*(IA*X),Y*sig,Z*sig
                    for n in range(Angle[m]): #loop over points in one pulse
                        x = Xs #x-field felt by DSS
                        y = -0.5*(cA[n]*Y-sA[n]*Zs) #y field
                        z = wDSS+(cA[n]*Z+sA[n]*Ys) #z field
                        theta = np.sqrt(x**2+y**2+z**2) #the rest in loop serves
                        #to calculate the rotation of DSS
                        a,t = np.cos(theta*0.5),-np.sin(theta*0.5)/theta
                        at2,t2 = 2*a*t,t*t
                        a2 = a*a
                        b2,c2,d2 = t2*x*x,t2*y*y,t2*z*z
                        ab,ac,ad = at2*x,at2*y,at2*z
                        bc,bd = 2*t2*x*y,2*t2*x*z
                        cd = 2*t2*y*z
                        mxp = (a2+b2-c2-d2)*mxm+(bc+ad)*mym+(bd-ac)*mzm
                        myp = (bc-ad)*mxm+(a2-b2+c2-d2)*mym+(ab+cd)*mzm
                        mzp = (bd+ac)*mxm+(cd-ab)*mym+(a2-b2-c2+d2)*mzm
                        mxm,mym,mzm = mxp,myp,mzp
                    Ap = Angle[m]*pi1*sig
                    Y,Z = np.cos(Ap)*Y-np.sin(Ap)*Z,np.cos(Ap)*Z+np.sin(Ap)*Y #
                    #update MH2O ofter each rf pulse (purely driven by rf)
                    c += Angle[m]
            MDSS[0,i,k+1],MDSS[1,i,k+1],MDSS[2,i,k+1] = mxp,myp,mzp #store MDSS
            #after each cycle
    return MDSS



def main():
    B0 = pa.B0            #main field (minus sign for positive gyrom.)
    vDSS = pa.vDSS        #chemical shift difference with H2O (ppm)
    mixing = pa.mixing    #spin-lock seq.  "Dipsi2","Garp","cw" or "Waltz16"
    tau90 = pa.tau90      #duration 90 pulse spinlock
    maxcyc = pa.maxcyc    #maximum number of cycles spin-lock
```

```
    ngrid = pa.ngrid      #H20 magnetization is phasemodulated number of phases
    wDF = 2*np.pi*pa.vDF  #dipolar field amplitude
    iniDSS = pa.iniDSS    #inital DSS magn. "iks", "why","zed" or "MH2O"

    order = pa.order      #lists of initial orders, SQ, DQ, TQ...(+ or -)
    plot = pa.plot        #True or False
    plotopt = pa.plotopt  #Dictionary of plotoptions (True or False):
                          #plane, planeabs,z, zabs
    plotcol = pa.plotcol  #colors plot (each order has its color)
    savedata = pa.savedata#if True save data to text file

    #sequences consist of a sequence of pulses of rotation angle integer/unit
    #always along the x-axis, with positive or negative sign (0, 180),
    #repated in a supercycle (e.g. for Dipsi2 a full cycle is 4*9= 36 pulses)
    if mixing=="Dipsi2":
        unit = 1
        Phase =  np.array([0,180,0,180,0,180,0,180,0])*(-1./90)+1
        Angle  = np.array([320,410,290,285,30,245,375,265,370])
        SupCyc = np.array([0,180,180,0])*(-1./90)+1
    if mixing=="Waltz16":
        unit = 1
        Phase =  np.array([0,180,0])*(-1./90)+1
        Angle  = np.array([90,180,270])
        SupCyc = np.array([0,0,180,180,180,0,0,180,
                       180,180,0,0,0,180,180,0])*(-1./90)+1
    if mixing=="Dipsi20": #to test unit = 10,  gives same result a Dipsi2
        unit = 10
        Phase =  np.array([0,180,0,180,0,180,0,180,0])*(-1./90)+1
        Angle  = np.array([3200,4100,2900,2850,300,2450,3750,2650,3700])
        SupCyc = np.array([0,180,180,0])*(-1./90)+1
    if mixing=="Garp":
        unit = 10
        Phase =  np.array([0,180,0,180,0,180,0,180,0,180,0,180,0,180,
                       0,180,0,180,0,180,0,180,0,180,0])*(-1./90)+1
        Angle  = np.array([305,552,2578,2683,693,622,850,918,1345,2561,
                       664,459,255,727,1195,1382,2584,649,709,772,
                       982,1336,2559,656,534])
        SupCyc = np.array([0,180,180,0])*(-1./90)+1
    if mixing=="cw":
        unit = 1
        Phase =  np.array([0])*(-1./90)+1
        Angle  = np.array([360*1]) #multiples of 360
        SupCyc = np.array([0])*(-1./90)+1

```

```
    tau1 = tau90/(90*unit) #duration of 1 step in calculation
    w1 = 2*np.pi/(4.*tau90) #rf amplitude

    H2Oini = np.array([[np.cos(i*2*np.pi/ngrid),np.sin(i*2*np.pi/ngrid),0]
                      for i in range(ngrid)]).T #initial condition solvent
    dt_sc = tau1*np.sum(Angle)*len(SupCyc) #lenght one supercycle
    time = dt_sc*range(maxcyc+1)#time from 0 to end mixing, in super cycle inc.
    print('Maximum mixing time:',time[-1])

    #next 3 lines. Evolution solvent after each supercycle. To a good
    #approximation a rotation arount the x-axis, with angular freq.=
    #-(3/4)*wDF*Mx(0)
    c = np.cos(-(3/4)*wDF*np.outer(H2Oini[0],time))
    s = np.sin(-(3/4)*wDF*np.outer(H2Oini[0],time))
    MH2O = np.array([np.outer(H2Oini[0],np.ones(maxcyc+1)),
                     np.einsum('i,ij->ij',H2Oini[1],c)-
                     np.einsum('i,ij->ij',H2Oini[2],s),
                     np.einsum('i,ij->ij',H2Oini[2],c)+
                     np.einsum('i,ij->ij',H2Oini[1],s)])

    MDSS = np.zeros_like(MH2O) #next lines initial MDSS
    if iniDSS == "MH2O":
        MDSS[:,:,0] = MH2O[:,:,0].copy()
    if iniDSS == "iks":
        MDSS[0,:,0]+=1.0
    if iniDSS == "why":
        MDSS[1,:,0]+=1.0
    if iniDSS == "zed":
        MDSS[2,:,0]+=1.0

    rfA  = w1*tau1 #rotation angle rf in 1 time-increment
    wDSS  = tau1*vDSS*B0*2*np.pi #rotation angle offset
    IA  = tau1*wDF #rotation angle wDF/MH2Ox around x-axis
    #next two lines, mixing factors for y and z contributions df (makes very
    #little difference to add 0.5 or not). This boils down to a precalculation
    #of sin and cos factors for MH2O evolution during pulses
    cA  = tau1*wDF*np.cos((np.arange(max(Angle))+0.5)*np.pi/(180*unit))
    sA  = tau1*wDF*np.sin((np.arange(max(Angle))+0.5)*np.pi/(180*unit))

    lA = len(Angle)
    lS = len(SupCyc)

    print('Compilation and parallel calculation starts')
    MDSS = evolveDSS(MDSS,ngrid,maxcyc,MH2O,SupCyc,Angle,Phase,IA,sA,
```

```
                     cA,rfA,wDSS,lA,lS,unit)
    #plot for different pfgs before acquistion. 'z' corresponds to, spoil-90
    #before the same pfg.
    c = 0
    for o in order:
        co = np.cos(o*2*np.pi*np.arange(ngrid)/(ngrid))/ngrid
        si = np.sin(o*2*np.pi*np.arange(ngrid)/(ngrid))/ngrid
        MDSSbuX = co.dot(MDSS[0])-si.dot(MDSS[1])
        MDSSbuY = co.dot(MDSS[1])+si.dot(MDSS[0])
        MDSSbuZx  = co.dot(MDSS[2])
        MDSSbuZy =  si.dot(MDSS[2])
        if plot:
            if plotopt['plane']:
                plt.figure('plane')
                plt.plot(time,MDSSbuX,color = plotcol[c])
                plt.plot(time,MDSSbuY,color = plotcol[c],linestyle='--')
            if plotopt['planeabs']:
                plt.figure('planeabs')
                plt.plot(time,np.sqrt(MDSSbuY**2+MDSSbuX**2),color = plotcol[c])
            if plotopt['z']:
                plt.figure('z')
                plt.plot(time,MDSSbuZx,color = plotcol[c])
                plt.plot(time,MDSSbuZy,color = plotcol[c],linestyle='--')
            if plotopt['zabs']:
                plt.figure('zabs')
                plt.plot(time,np.sqrt(MDSSbuZx**2+MDSSbuZy**2),color = plotcol[c])
        c +=1
        #can be saved to make own plots, especially to compare
        if savedata:
            np.savetxt('mag'+str(o)+'.txt',[time,MDSSbuX,MDSSbuY,MDSSbuZx,
                                            MDSSbuZy])
    plt.show()
if __name__ == '__main__':
    main()
```
